# Prevalence and Associations of geriatric depression in Sudur-Paschim Province of Nepal: A community-based cross-sectional study

**DOI:** 10.1192/j.eurpsy.2024.1314

**Published:** 2024-08-27

**Authors:** D. Kunwar

**Affiliations:** psychiatry, kathmandu university school of medicine, kathmandu, Nepal

## Abstract

**Introduction:**

The elderly population is rapidly growing worldwide. Depression is an important public health concern among the elderly population in terms of its prevalence and the burdens of ill health in individual sufferers and families.

A considerable number of studies focusing on the prevalence, associated factors, and treatment of depression have been conducted in Western Europe and North America. However, this kind of research is relatively limited in Nepal.

**Objectives:**

- To find out and prevalence and its associated factors of depression among the elderly in sudur paschim province Nepal.

**Methods:**

This community-based, cross-sectional, door-to-door survey was conducted in the two representative districts of Sudur-paschim Province, Nepal. Eligible participants were assessed for geriatric depression and quality of life. the sample size was calculated at 945.

**Results:**

Depression was found in 43.9% of the participants.

The association of different variables with WHOQOL-8 first question. Income was significantly associated with poor quality of life (p-value 0.04).

Furthermore, we have evaluated the association of different variables across four domains of the WHOQOL-8 scale and found statistically significant differences across age, sex, residence, occupation, income, history of smoking, history of alcohol use, comorbidities, and depression.

**Conclusions:**

Depression is highly prevalent among the elderly in the Sudur-Paschim Province of Nepal. So policymakers should take this research outcome seriously and should keep the mental health of the elderly population a priority during the implementation of public health policies.

**Disclosure of Interest:**

None Declared

